# Serum Metabolic Profile in Patients With Long-Covid (PASC) Syndrome: Clinical Implications

**DOI:** 10.3389/fmed.2021.714426

**Published:** 2021-07-22

**Authors:** Evasio Pasini, Giovanni Corsetti, Claudia Romano, Tiziano M. Scarabelli, Carol Chen-Scarabelli, Louis Saravolatz, Francesco S. Dioguardi

**Affiliations:** ^1^Division of Cardiac Rehabilitation, Scientific Clinical Institutes Maugeri Istituto di Ricovero e Cura a Carattere Scientifico (IRCCS), Lumezzane, Italy; ^2^Division of Human Anatomy and Physiopathology, Department of Clinical and Experimental Sciences, University of Brescia, Brescia, Italy; ^3^Center for Heart and Vessel Preclinical Studies, St. John Hospital and Medical Center, Wayne State University, Detroit, MI, United States; ^4^Division of Cardiology, Richmond Veterans Affairs Medical Center, Richmond, VA, United States; ^5^Department of Medicine at St. John Hospital, Wayne State University, Detroit, MI, United States; ^6^Department of Internal Medicine, University of Cagliari, Cagliari, Italy

**Keywords:** SARS-CoV2, Long-COVID-syndrome, PASC, ferritin, d-dimer, COVID-19, coagulation, rehabilitation

## Abstract

**Background:** Many patients who have been suffering by Covid-19 suffer of long-Covid syndrome, with symptoms of fatigue and muscular weakness that characterize post-acute sequelae SARS-CoV-2 infection (PASC). However, there is limited knowledge about the molecular pathophysiology, and about the serum profile of these patients.

**Methods:** We studied the blood serum profile of 75 selected patients, with previous confirmed Covid-19, 2 months after hospital discharge, who reported new-onset fatigue, muscle weakness and/or dyspnea not present prior to the virus infection and independently from concomitant diseases and/or clinical conditions.

**Results:** All patients had very high serum concentrations of ferritin and D-Dimer. 87 and 72% of patients had clinically significant low levels of hemoglobin and albumin, respectively. Seventy three percentage had elevations in erythrocyte sedimentation rate and CRP. Twenty seven percentage had elevations in LDH.

**Conclusions:** The co-existence of patient symptoms along with blood markers of coagulation, protein disarrangement and inflammation suggests ongoing alterations in the metabolism, promoting an inflammatory/hypercatabolic state which maintains a vicious circles implicated in the persistence of PASC. The persistence of altered D-Dimer levels raises the possibility of long-term risks of thromboembolic disease. All these markers levels should be accurately evaluated in the long-term follow-up, with individualized consideration for prophylactic nutritional, anti-inflammatory and/or anticoagulant therapy if indicated.

## Highlights

- Fatigue and dyspnea are the more frequent symptoms of the Long-COVID.
- Patients shows higher serum ferritin, D-Dimer, CRP, and lower hemoglobin and albumin.
- Persistent metabolic alterations maintains an hypercatabolic state.
- Higher D-Dimer raises the possibility of risks of thromboembolic disease.
- Prophylactic nutritional and anti-coagulant/inflammatory therapies should be evaluated.

## Introduction

Severe acute respiratory syndrome coronavirus-2 (SARS-CoV2) is an enveloped mRNA beta-coronavirus causing coronavirus disease 2019 (Covid-19). Covid-19 infection has risen to a global pandemic with important sanitary and socio-economic consequences (1). The clinical and pathological features of acute infection have been extensively described and several clinical and/or laboratory markers have been proposed to predict the disease's severity and associated morbidity. Indeed, studies show that blood levels of pro-inflammatory markers as interleukins (IL-6, IL-10), TNF-α, C-reactive protein (CRP), D-Dimer (DD), lactate dehydrogenase (LDH) and/or prealbumin are associated with severe Covid-19 and they correlate with patients' survival (2–5).

Interestingly, several recent studies show that 50–70% of patients still experience a variety of symptoms up to 2–6 months following total recovery from the acute phase of Covid-19 infection (6, 7). This clinical condition is named “Long-Covid” or “Coronavirus Long Haulers” (8), defined as a condition in which coronavirus symptoms persist after the virus has left the body. Recently, experts have coined a new term for it: post-acute sequelae SARS-CoV-2 infection (PASC). Although fatigue, muscular weakness and dyspnea are the more frequent symptoms of the PASC syndrome with consequent reduction of the patient's quality of life, the presence of other types of symptoms suggest that many organs are involved (6, 7). However, there is limited knowledge on the molecular mechanisms implicated in this syndrome (6).

To date, it has not been established whether elevated serum markers in the acute phase remain persistently increased in the PASC syndrome. Consequently, considering the mechanism of viral replication based on subversion of host protein metabolism with massive cell damage, we have postulated that serum markers may be useful tools in the identification of impaired human cellular function in both the acute phase, as well as the expression of prolonged cell metabolic impairment present in PASC (9).

The aim of this study was to investigate the serum metabolic profile in selected patients with PASC syndrome who complained of fatigue, muscular weakness and dyspnea in the absence of pulmonary disease or other confounding diseases which may account for the symptoms. Fatigue was assessed using both subjective and objective methods.

## Methods

This study is a prospective cohort study of previously hospitalized patients with laboratory confirmed mild-moderate Covid-19 disease. We studied 75 selected patients (42 males and 33 females), after 60 days from hospital discharge, who reported new-onset fatigue not present prior to the virus infection. Patients with symptoms of fatigue were seen in our private ambulatory setting from July to September 2020. Based on the fundamental guidance of “good medical practice” (www.gmc-uk.org) we performed tests and blood analyses according to professional and ethical standards. For these reasons, ethical approval was not required under local legislation. Written informed consent was obtained from all patients for blind management of the data.

In an effort to exclude any potentially confounding factors, we studied selected patients with fatigue, muscle weakness and/or dyspnea independently from concomitant diseases and/or clinical conditions which could influence physical performance, including thrombotic episodes.

Clinical data and demographic characteristics were collected from the patients' electronic medical records. At the follow up visit, all patients were interviewed face-to-face. Specific standardized and validated self-reported symptoms and the Fatigue Assessment Scale (FAS) were administered (10). The FAS scoring range is 10–50 with two categories; FAS score 10–21: no fatigue (normal); FAS score 22–50: substantial fatigue.^1^ In addition, the presence of muscular weakness was objectively evaluated by use of the 1-min sit to stand (STS) test as previously described (11). We limited follow-up to only very selected patients with subjective reported symptom of fatigue (FAS score >22) and objective data of muscular weakness (defined as the number of repetitions <70% of the predicted values at STS-test, using the normal values according to patients' age and sex, but with normoxia (>94%) at rest, absence of oxygen desaturation (<4% of rest) during STS-test to avoid exercise intolerance due to oxygen desaturation, exercise-induced, and without concomitant previous presence of inflammatory, endocrine, cognitive, orthopedic and/or neurological disorders, or reported fatigue or muscular weakness. Venous blood samples were collected from these patients and hematological data were quantified. Due to gender differences in laboratory reference values, we stratified results in tables according to gender. The “normal range” values are specific to the age group studied.

Additional exclusion criteria were: major cardiac arrhythmias and hemodynamics instability, enable to sitting down independently, and FAS score <22.

### Statistical Analysis

Patient's clinical data were presented as mean ± standard deviation or expressed as absolute values with percentages of total. Hematological data were expressed as mean ± standard deviation and Welch's *t*-test was applied to compare means. The normality of distribution data was assessed by Shapiro-Wilk Test (https://www.statskingdom.com).

## Results

**Figure 1:**
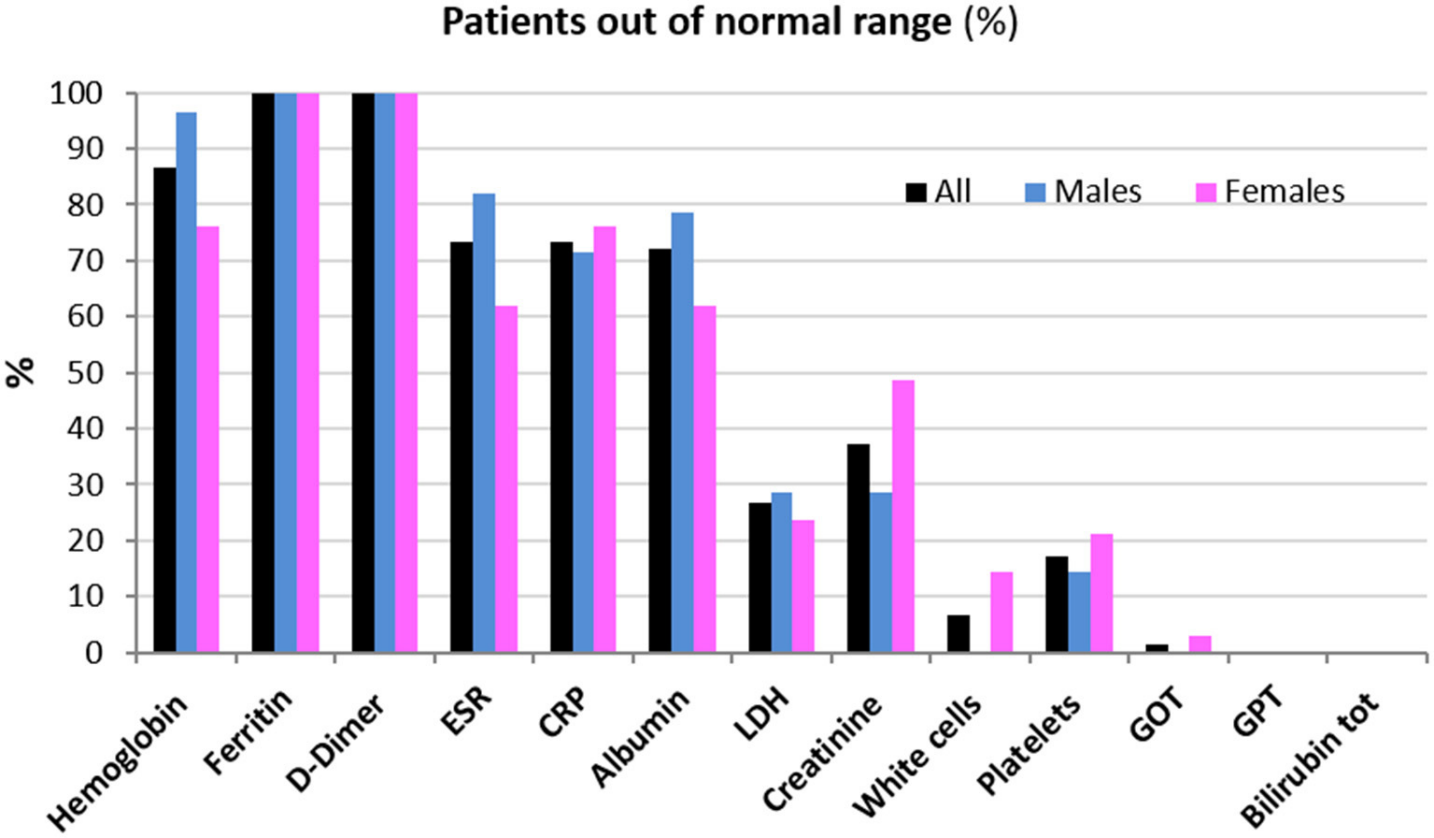
Patients (%) whose hematological data are out of the normal range 60 days from hospital discharge.

Clinical data of patients, including length of hospitalization (from diagnosis to discharge) and time from hospital discharge are presented in Table 1. The mean length of hospitalization was about 20 days for both genders. During hospitalization, 52% of patients (about 45% males and 60% females) required oxygen therapy. The mean time from hospital discharge was about 60 days, with no difference between genders and between patients with/without oxygen supplementation and non-invasive ventilation. Over 90% of patients had a normal BMI. In all patients, steroid therapy was started on the day of hospital admission and maintained with tapering doses until discharge. Blood biochemical data from all patients are presented according to gender in Table 2. Except for higher ESR levels in males, there were no differences in the blood data between the two gender groups. However, the percentage of patients with values that do not fall within the normal range, specific to the age group studied, is of considerable importance. Indeed, the data demonstrated that all patients (100%) had very high serum concentrations of ferritin and DD. In addition, many patients had clinically significant low levels of hemoglobin (87% of patients) and albumin (72%), and elevations in erythrocyte sedimentation rate (ESR) (73%), and CRP (73%) (Figure 1). The comparison of main hematological data from all patients at hospital admission (acute phase) and 60 days from hospital discharge are resumed in Table 3.

**Table 1:** Patient's clinical data according to gender.

	**All**	**Male**	**Female**	***p***
Normality of distribution = W (range 0–1)	0.980	0.967	0.963	–
Age (y.o.), mean (SD)	72 (7)	71 (8)	73 (6)	0.24
Days from discharge, mean (SD)	60 (5)	60 (6)	61 (5)	0.44
Days of hospitalization, mean (SD)	20 (9)	19 (10)	21 (9)	0.37
Hospitalized in medical service, No. (%)	75 (100)	42 (100)	33 (100)	–
Supplemental oxigen, nasal cannula No. (%)	39 (52)	19 (45)	20 (61)	0.17
Non invasive ventilation (NIV), No. (%)	6 (8)	4 (9.5)	2 (6)	0.58
Antibiotics, No. (%)	63 (84)	35 (83)	28 (85)	0.82
Corticosterioids, No. (%)	32 (43)	22 (52)	10 (30)	0.06

*The normality of distribution data (W) was assessed by Shapiro-Wilk Test. Data of time from hospital discharge and time of hospitalization, are expressed as days (SD). Other data are expressed as number and (%). The T-test was used to compare differences in means, and comparison of proportions test was performed to compare differences between the men and women (MedCalc software)*.

**Table 2:** Hematological data from all patients and according to gender after 60 days from hospital discharge.

	**All**	**Males**	**N.r. (males)**	**Females**	**N.r. (females)**	**T**	***P***
Hemoglobin, g/dL	11.23 (1.55)	11.5 (1.42)	14–18	10.9 (1.68)	12–16	1.67	0.1
Ferritin, ng/mL	496.24 (288.2)	474.2 (210.6)	22–275	525.6 (371.3)	5–204	0.71	0.48
D-Dimer, ng/mL	900.71 (350.5)	931.4 (366.9)	<500	859.8 (331.6)	<500	0.88	0.38
ESR, mm/h	30.27 (18.73)	35.82^*^ (19.3)	<15	22.86 (15.45)	<15	3.23	0.001
CRP, mg/L	9.12 (4.8)	9.92 (5.7)	<5	8.06 (2.9)	<5	1.83	0.07
Albumin, g/L	33.04 (3.37)	32.62 (3.04)	35–55	33.6 (3.77)	35–55	1.21	0.23
LDH, U/L	197.74 (33.35)	199.13 (37.6)	125–220	196.05 (28.3)	125–220	0.40	0.69
Creatinine, mg/dL	0.68 (0.21)	0.70 (0.23)	0.7–1.2	0.67 (0.16)	0.6–1.1	0.66	0.51
White cells, 10^3^/uL	6.4 (1.83)	6.74 (1.9)	4–10	5.94 (1.71)	4–10	1.91	0.06
Platelets, 10^3^/uL	250.63 (127.1)	235.76 (93.2)	150–450	269.55 (159.9)	150–450	1.08	0.29
GOT, U/l	21.35 (7.32)	20.9 (7.5)	≤ 34	21.95 (7.23)	≤ 34	0.61	0.54
GPT, U/l	27.45 (13.12)	26.15 (11.85)	≤ 55	29.11 (14.7)	≤ 55	0.94	0.35
Bilirubin tot, mg/dL	0.5 (0.17)	0.45 (0.13)	0.2–1.2	0.51 (0.19)	0.2–1.2	1.55	0.13

*D-Dimer is expressed as Fibrinogen Equivalent Units (FEU). ESR, erythrocyte sedimentation rate. CRP, C-reactive protein. LDH, lactate dehydrogenase. Data are expressed as mean (SD). N.r., normal range values. Welch's t-test: male vs. females*

* *p < 0.05*.

**Table 3:** Comparison of main hematological data from all patients at hospital admission (Covid-19 acute phase) and 60 days from hospital discharge (final).

	**Admission**	**Final**	**T**	***p***
Hemoglobin, g/dL	10.5 (1.6)	11.23 (1.55)^*^	2.84	0.005
Ferritin, ng/mL	624.9 (170.1)	496.24 (288.2)^*^	3.33	0.001
D-Dimer, ng/mL	1,350 (447.6)	900.71 (350.5)^*^	6.84	0.000
ESR, mm/h	47.5 (26.1)	30.27 (18.73)^*^	4.64	0.000
CRP, mg/L	12.75 (5.6)	9.12 (4.8)^*^	4.26	0.000
Albumin, g/L	30.9 (2.1)	33.04 (3.37)^*^	4.67	0.000

*ESR, erythrocyte sedimentation rate. CRP, C-reactive protein. Data are expressed as mean (SD). N.r., normal range values. Welch's t-test*,

* *p < 0.05*.

## Discussion

This study demonstrates that, at 2 months after hospital discharge for acute Covid-19 infection, selected patients with reported fatigue and muscular weakness independent from concomitant diseases continued to exhibit elevated indexes of altered cell proteins machinery as ferritin, hemoglobin and albumin, as well as inflammatory markers as CRP, ESR, LDH and marker of activated coagulation as DD, suggesting ongoing impairment in the global metabolism of affected patients. Covid-19 replication and Covid-19-induced metabolic disarrangements are discussed in further detail in the following section.

### SARS-CoV2 Replication

It is well-known that SARS-CoV2 is obligate intracellular pathogen which cannot replicate without macromolecules and metabolic machinery of the host cell. The spike proteins of the virus bind receptors identified as angiotensin-converting enzyme-2 (ACE2) present on the cell membranes of many organs. This binding causes conformational changes of the viral capsid and its fusion with the cellular membrane and release of the viral genome inside the host cell. The viral nucleic acid (mRNA) starts to synthesize the encoded proteins using the host cell ribosomes and needs of molecules, mainly proteins, and energy of the infected cell in order to replicate the viral genome and synthetize his progeny. Then, the newborns virions are released from the host cell (9, 12).

Given that the virus *per se* hijacks and alters cellular metabolism to fulfill its needs for replication, it predominantly targets the amino acids (AAs) to meet such needs. Indeed, AAs are vital biomolecules for viral protein synthesis and are precursors for viral nucleotide metabolism because they are the unique molecules with the ability to provide nitrogen (13). It is well-established that AAs are the building blocks of cell proteins. Consequently, the virus implements metabolic strategies to dissemble cellular proteins, thereby activating apoptosis and autophagy so that the resulting AAs are available for its metabolic needs.

### Cell Apoptosis and Autophagy SARS-CoV2-Induced

Although apoptosis and autophagy are two distinct cell self-destructive processes, their regulation is connected, with the same regulators sometimes controlling both of them (14).

Apoptosis is characterized by a series of dramatic perturbations that contribute to demolish cellular molecules and architecture, causing the cell death. It occurs in damaged cells by activation through disease or noxious agents (15).

Autophagy is a process in which cells digest their own cytoplasmic organelles and/or molecules when stimulated by specific stimuli. The resulting products can be recycled to generate energy and build new proteins according to specific molecular information (16).

Apoptosis and autophagy are primarily stimulated by inflammatory molecules, as interleukins caused by SARS-CoV2-induced inflammation (17). Maintenance of inflammation is confirmed by the presence of high levels of CRP still detectable in affected patients. Indeed, CRP is a molecule synthesized by the liver which senses the balance between pro- and anti-inflammatory cytokines, so that CRP rise indicates predominance of pro-inflammatory interleukins (18). Our study supports the presence of persistent inflammation and cellular damage based on continued elevation in LDH levels in more than 27% of our patients.

Although elevated levels of LDH are nonspecific since it is widely distributed in tissues of the body, particularly in the kidney, heart, skeletal muscle, brain, liver and lungs, and we had no access to evaluation of isoenzymes, LDH modifications should be considered markers of cellular distress (19, 20).

Recent data show that virus directly stimulates apoptosis. Interestingly, SARS-CoV2 spike (S), nucleocapsid (NP), envelope (EP), proteins, as well as nonstructural viral proteins named OFR-6, 7 s and 9b proteins are pro-apoptotic in the host cell (21–24). In detail, SARS-CoV2 M activates cellular apoptosis-stimulating molecules as caspase-8 and−9 (25) and that viral nonstructural protein 7a induces mitochondrial damage by altering outer membrane permeability with consequent release of Cytochrome-C which is the main signaling for mitochondrial-mediated apoptosis (26).

In addition, data suggest that phosphorylation of MAPKs cascade occurs in the SARS-CoV2 infected cells (27). MAPKs are a group of evolutionary well-conserved kinases which are activated by environmental stresses induced by viral infection. They regulate many cellular processes including catabolic pathways such as autophagy, but also mRNA translation which is fundamental for viral replication (28, 29).

The presence of virus-activated inflammation and cellular stress promoting an hypercatabolic/hypermetabolic state was also confirmed by our finding of a significant reduction in circulating albumin (30). Furthermore, low hemoglobin levels were detected in our study patients. Many cell proteins are hemeproteins containing an iron atom essential for the enzymatic activities in cell homeostasis; blood hemoglobin is the most recognized and easiest to measure hemeprotein.

Notably, hemeproteins include many other fundamental proteins for the cell's metabolism including: myoglobin, catalase, cyclooxygenase, peroxidase, cytochrome p450 and nitric oxide synthase, and the mitochondrial proteins responsible for energy production and maintenance of cell metabolism that are likely reduced by Covid-19 (31). Moreover, a nonstructural protein of SARS-CoV2, named ORF3a, may have a direct link capable of digesting the heme group of mitochondrial Cytochrome-C, thereby dissociating the iron of heme from the protein ring shape pyrroles (32). Consequently, the demolition of heme proteins causes important impairment of cellular metabolic pathways, mitochondrial dysfunction and iron dysmetabolism (33).

In summary, the virus invades the host cells, gaining control over cellular metabolism, activating catabolic pathways such as apoptosis and/or autophagy which demolish predominantly the cell proteins to make AAs available for virions production. This virus strategy results in cellular damage, protein dysfunction, mitochondrial energetic loss and iron leakage with subsequent serum ferritin increase and patient-reported fatigue and objective muscular weakness.

### Hyper-Ferritinemic Syndrome

Hyper-ferritinemic syndrome, found in the study patients, is a complex phenomenon which may facilitate cellular apoptosis. Interestingly, the presence of iron metabolic disarrangement in SARS-CoV2 infection may not only be secondary to digestion of hemoproteins with resultant iron cell leakage, but consequential to indirect and direct metabolic stimulation by virus infection (34).

Recent data suggest that serum ferritin levels rose in response to inflammation concomitantly with the virus-induced increase of cytokines as IL-1ß and IL-6, primarily because these molecules stimulate the synthesis of hepcidin, a protein that regulates the entry of iron into the circulation in mammals (35). Interestingly, recent research report that SARS-CoV2 has intrinsic hepcidin-mimetic action which increases hyper-ferritinemic syndrome (36).

Consequently, the SARS-CoV2-induced alteration of iron metabolism reinforced cellular damage because iron overload leads to cell apoptosis, a process called ferroptosis, with enhanced oxidative stress and lipid-peroxidation which, in turn, consolidate cellular protein disarrangements and increase mitochondrial dysfunction with additional release of Cytochrome-C (37). These conditions create a vicious cycle by which the virus continues to digest cellular proteins to make molecules available for its replication.

### Link Between Inflammation, Hyper-Ferritinemic Syndrome and High D-Dimer

In our study cohort, DD was persistently elevated in PASC syndrome. Indeed, it is well-established that inflammation stimulates coagulation (38). What is less clear is whether mitochondrial abnormalities caused by SARS-CoV2 infection can reinforce activation of the coagulation cascade.

We know that platelets are blood components with a fundamental role in thrombosis. Indeed, platelet adhesion, activation and aggregation stimulate coagulation factors and other mediators to achieve hemostasis with blood clot formation. Interestingly, DD is a breakdown product of cross-linked fibrin by the action of Plasmin protein, not produced by action of plasmin on unclothed fibrinogen, and therefore specific for fibrin, confirming that both Thrombin and Plasmin generation have occurred. This test is used in the diagnosis of disseminated intravascular coagulation and to screen for venous thrombosis and acute myocardial infarction since DD are not produced by action of Plasmin on unclothed fibrinogen. Therefore, DD are involved in coagulation cascade and its increase in the blood is a marker of hypercoagulability (39). However, in this contest, it is important to remember that platelets are non-nucleated cell, so their function is regulated by their mitochondria (33, 40). Consequently, SARS-CoV2-induced mitochondrial dysfunction in platelets could create metabolic dishevelment able to activate the coagulation cascade with resulting DD elevations (33). In addition, we must consider that platelet activation, with subsequent activation of coagulative proteins and DD formation, is a marker of Covid-19 likely dependent of MAPKs pathway activation as already discussed (27). Taken together, the aforementioned phenomenon could explain presence of elevated DD in our patients.

**Figure 2:**
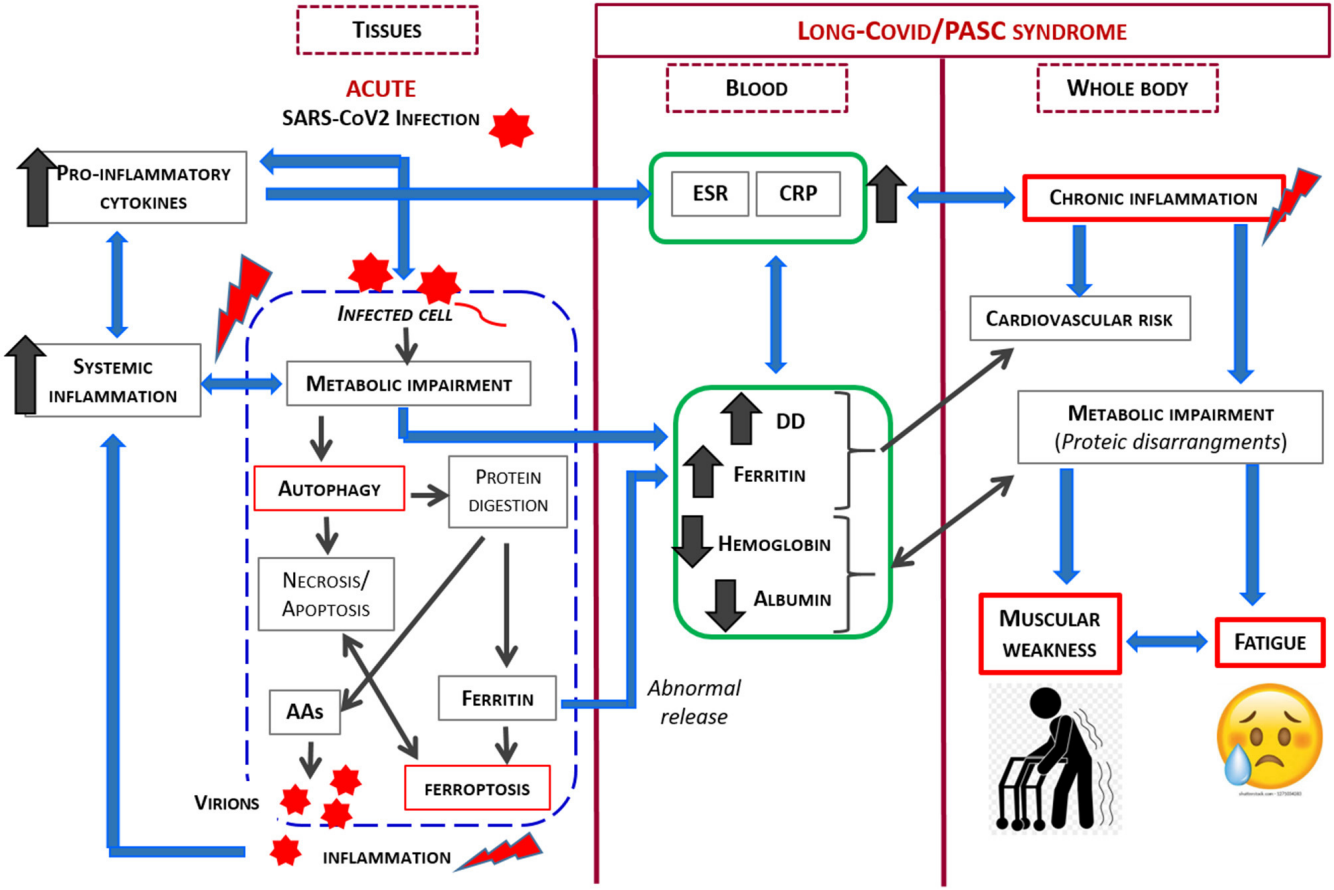
Schematic representation of the link between SARS-CoV2 infection and metabolic impairment in infected cell and Long-Covid/PASC syndrome.

Considering the previously described phenomenon, the relationship between SARS-CoV2 and its host is complex, and is influenced by many factors. However, as SARS-CoV2 is an obligate parasite which must utilize the cell's metabolic machinery to replicate, the virus has evolved strategies to maximally utilize the host resources. In light of the current knowledge, our study demonstrates that the co-existence of patient symptoms along with blood markers of inflammation and protein/coagulation disarrangement several months after the acute virus infection, strongly suggests ongoing alteration in the host metabolism, with this alteration implicated in the persistent signs and symptoms. The link between SARS-CoV2 infection and metabolic impairment in infected cell and PASC syndrome is shown in Figure 2.

### Limits of the Study

One of the major limitations of our study is whether the altered serum variables, and/or muscular weakness pre-existed prior to the SARS-CoV2 infection. Although we took precautions by limiting study enrollment to patients without concomitant diseases which may affect the blood variables, and patients who reported fatigue only after viral infection, we cannot exclude that the concomitant presence of comorbidities may contribute to worsening symptoms and signs of PASC syndrome.

Secondly, we studied patients with a mean age of 72 ± 7 years old. Studies suggest that aging could cause a phenomenon called “Inflamm-aging” characterized by an aging-induced low-grade persistent increase in inflammatory molecules. Aging-induced in inflammatory molecules. However, the clinical effects and pathophysiological significance of this phenomenon remain to be elucidated. Moreover, it has been suggested that an aged immune system is plastic and able to neutralize or block cytokine action by specific molecules (41). Consequently, the findings may be due to long-lasting virus effects.

### Clinical Implication

Taking into consideration the possible pathophysiology of PASC syndrome as illustrated, the goals of pharmacological therapy for these patients should be aimed at (i) early restoration of the metabolic cell's molecules and machinery providing proteins and/or AAs with enzymatic cofactors as anabolic vitamins and ions to stimulate protein synthesis in order to restore the body metabolism, and (ii) activation of the cell's anabolic metabolism by personalized physical rehabilitation as we have previously demonstrated in patients with chronic diseases (42).

We observed consequences of a protracted hypermetabolic-hypercatabolic condition in our patients, easily detected both by physical exam and laboratory findings. Correction of these needs by optimizing nutritional care and supporting personalized nitrogen intake to counteract inflammation and promote tissue repair (30), should be considered a first-line care.

An increase in DD correlates with mortality in severe Covid-19 infections (43), and although the clinical significance of elevated DD values has been recently reviewed and cautious interpretation is recommended (44), we postulate that the persistence of altered coagulation months after hospitalization should raise attention to the possible long term risks of thromboembolic disease in those patients, risks that have been discussed prevalently in acute settings along with the advantages and disadvantages of aggressive therapy (45). On the other hand, autopsies confirmed DD value in identifying the most serious Covid-19 dependent illnesses (46); the association between increased risk and high-dose steroid therapy has been observed (47). DD evaluation should be included in long-term follow up of patients, particularly in older patients with co-morbidities which increase the risk of thromboembolic diseases. Consideration of appropriate prophylactic anticoagulant therapy should be discussed with medical experts, along with the patient and the patient's caregiver to promote shared decision-making, and to minimize late mortality linked to PASC despite recovery from the acute phase.

## Conclusion

The study, based on simple and repeatable tests, provides additional insight on the different aspects of SARS-CoV2-induced complications. In particular, it helps to uncover a possible cause of PASC. Ongoing studies are essential to improve our understanding of the pathophysiology of this relatively new illness and its evolution in order to improve the treatment, and perhaps cure, of the SARS-CoV2 disease.

## Data Availability Statement

The raw data supporting the conclusions of this article will be made available by the authors, without undue reservation.

## Ethics Statement

Patients were seen in the private ambulatory setting. Based on the fundamental guidance of good medical practice (www.gmc-uk.org) we performed tests and blood analyses according to professional and ethical standard. Ethical review and approval was not required for the study on human participants in accordance with the local legislation and institutional requirements. The patients/participants provided their written informed consent to participate in this study. Informed consent for blind management of the data was obtained from all subjects involved in the study.

## Author Contributions

EP, GC, and FD: conceptualization. EP and GC: methodology, formal analysis, writing—original draft preparation, project administration, and investigation. GC and CR: software and data curation. FD, TS, and LS: validation. GC: resources. TS and CC-S: writing—review and editing. FD and LS: supervision. All authors contributed to the article and approved the submitted version.

## Conflict of Interest

The authors declare that the research was conducted in the absence of any commercial or financial relationships that could be construed as a potential conflict of interest.

## Footnotes

^1^https://www.wasog.org/educational-material/fatigue-assessment-scale.html (accessed February 26, 2021).
